# Synergistic effects of co-expression plasmid-based ADAM10-specific siRNA and GRIM-19 on hepatocellular carcinoma *in vitro* and *in vivo*

**DOI:** 10.3892/or.2021.8235

**Published:** 2021-11-26

**Authors:** Songyang Liu, Wei Zhang, Kai Liu, Yingchao Wang, Bai Ji, Yahui Liu

Oncol Rep 32: 2501-2510, 2014; DOI: 10.3892/or.2014.3503

Following the publication of the above paper, it was drawn to the Editor's attention by a concerned reader that various data featured in several of the figures were strikingly similar to data appearing in different form in other articles by different authors. An independent enquiry was launched by the Editorial Office, which revealed that cell migration assay data featured in Fig. 5A and C of the above article had been included in another article written by different authors submitted for publication some 12 months previously.

Owing to the fact that the contentious data in the above article were already under consideration for publication prior to its submission to *Oncology Reports*, the Editor has decided that this paper should be retracted from the Journal. After having been in contact with the authors, they agreed with the decision to retract the paper. The Editor apologizes to the readership for any inconvenience caused.

